# Dynamic changes in peripheral blood-targeted miRNA expression profiles in patients with severe traumatic brain injury at high altitude

**DOI:** 10.1186/s40779-019-0203-z

**Published:** 2019-04-30

**Authors:** Si-qing Ma, Xue-xia Xu, Zong-zhao He, Xin-hui Li, Jun-ming Luo

**Affiliations:** 1 Department of Intensive Care Unit, Qinghai Provincial People’s Hospital, Xining, 810007 China; 2 Department of Pathology, Qinghai Provincial People’s Hospital, Xining, 810007 China

**Keywords:** Severe traumatic brain injury, miRNA expression profile, High altitude

## Abstract

**Background:**

The aim of this work is to detect and compare the peripheral blood miRNA expression profiles in patients with severe traumatic brain injury (sTBI) 2, 12, 24, 48, and 72 h after injury at high altitude and to predict the target genes of differential expressed miRNAs.

**Methods:**

Twenty sTBI patients from high-altitude areas were randomly selected according to the inclusion and exclusion criteria and were divided into five groups: the 2-h group, 12-h group, 24-h group, 48-h group, and 72-h group. Peripheral blood miRNA expression profiles were detected using real-time quantitative PCR (qRT-PCR).

**Results:**

The expression levels of miR-18a, miR-203, miR-146a, miR-149, miR-23b, and miR-let-7b in peripheral blood showed significant differences between the 2-h group and the 12-h group. The expression levels of miR-203, miR-146a, miR-149, miR-23b, and miR-let-7f in peripheral blood were up-regulated in the 24-h group. In the 48-h group, the expression levels of miR-181d, miR-29a, and miR-18b were upregulated. In the 72-h group, the expression levels of miR-203, miR-146a, miR-149, miR-23b, and miR-let-7f changed. The main target genes of the differentiation expressed miRNAs were genes that regulate inflammatory responses, apoptosis, and DNA damage/repair.

**Conclusions:**

miRNAs may be involved in the pathogenesis of sTBI by dynamically regulating the target genes that regulate inflammatory responses, apoptosis, and DNA damage/repair pathways.

## Background

Severe traumatic brain injury (sTBI) is the most common accidental injury seen in emergency departments, such as intensive care units (ICUs) [1]. sTBI has a high mortality rate and can lead to different degrees of sensory-motor and cognitive dysfunction in surviving patients [2, 3]. Early diagnosis and accurate assessment of the severity of TBI not only save the lives of patients, but also are critical for the secondary prevention of various complications [4, 5].

MicroRNAs (miRNAs) are a class of endogenous small noncoding single-stranded RNA molecules [6]. Studies have shown that miRNAs can regulate gene expression levels during the development and progression of diseases under normal physiological conditions, and changes in miRNA expression profiles reflect alterations in physiological and pathological conditions [7]. Similarly, miRNAs play a very important regulatory role in the pathogenesis of sTBI [8]. The Qinghai-Tibet Plateau belongs to a high-altitude area, which can lead to hypoxia. The physiological and clinical manifestations of brain injury in a hypoxic environment can be even more severe. However, few reports have described sTBI at high altitude. Therefore, it is of great significance to explore the mechanisms that aggravate sTBI in high-altitude areas.

In the present study, we dynamically monitored the changes in miRNA expression profiles in the peripheral blood of sTBI patients in the acute phase (within 3 days) at 2, 12, 24, 48 and 72 h, in Xining, Qinghai Province, China, in an attempt to explore the change in miRNA expression profiles in high altitude locations under a hypoxic environment and provide new evidence for the development of molecular biological treatments and clinical therapeutic strategies for sTBI.

## Methods

This is a single-center, prospective study. The study protocol was approved by the Ethics Committee of Qinghai People’s Hospital, Xining, China. Written informed consent was obtained from each subject.

sTBI was defined as a Glasgow Coma Scale (GCS) score of 3–8 [9]. The inclusion criteria were as follows: a) sTBI patients receiving treatment in Xining and its surrounding areas (at 2000–3500 m above sea level); b) patients who presented at Qinghai People’s Hospital within 24 h of onset; c) both men and women aged 18–60 years; d) patients who survived within 72 h after injury; and e) patients who understood the objective of the study, and the family members of the patient voluntarily participated in the study by signing the informed consent form. The exclusion criteria were as follows: a) patients with a chronic disease; b) patients with severe comorbidities such as hypovolemic shock and severe thoracic/abdominal injuries; and c) patients younger than 18 years or older than 60 years.

The reagents and instruments used in this study included PAXgene RNA Tubes (BD, USA), miRNeasy Mini kit (Qiagen, USA), NanoDrop 2000 (Thermo, USA), RT2 miRNA PCR Arrays Human Mifinder (Qiagen, USA), 2720 Thermal Cycler (ABI, USA), 7500 Real-Time PCR System (ABI, USA), and a microphotometer (Imple, Germany).

Changes in miRNA expression profiles in 20 eligible sTBI patients 2, 12, 24, 48 and 72 h after admission were detected. For all these patients, 2.9 ml of peripheral blood samples was collected using a PAXgene Blood RNA Tube at the above indicated time points and stored in a − 80 °C cryogenic refrigerator for further use.

Dynamic differential miRNA expression levels in peripheral blood were detected in the acute phase. The miRNA expression levels were examined using the RT2 miRNA PCR Arrays Human Mifinder kit. According to the concentration of total miRNA, 100 ng of total miRNA was harvested from each sample to synthesize cDNA with the RT2 Easy First Strand Kit. Then, 100 μl of cDNA template, 1275 μl of 2X RT2 SYBR Green miPCR Master Mix and distillation-distillation H_2_O (ddH_2_O) were added to a final volume of 2550 μl. An equal volume of the mixture was dispensed into a 96-well plate. A 25 μl reaction system was prepared, and ABI7500 for PCR assay was used for 40 cycles of amplification. The raw data were exported using the ABI 7500 Sequence Detection System and then uploaded to Qiagen’s on-line analysis software (http://pcrdataanalysis.sabiosciences.com/pcr/arrayanalysis.php) to screen out differentially expressed miRNAs.

By using bioinformatic analysis methods, we predicted and analyzed the target genes of the differentially expressed miRNAs and their functions using online tools, including TargetScan, PieTar, and miRanda. Quantitative PCR was used to determine the expression levels of the target genes of differentially expressed miRNAs.

During the miRNA analysis, SA_miRNA_005 was used as an internal control. The differences in target miRNA expression levels between the study samples and the corresponding control were calculated by the 2^-ΔΔCt^ method (expressed as a ratio, and the target miRNA expression level of the control sample was set to 1), where ΔΔCt = ΔCt study sample - ΔCt control sample; ΔCt = Ct miRNA - Ct SA_miRNA_005; and 10 Ct is the number of amplification cycles at which a defined threshold of the real-time fluorescence intensity is reached when the amplification shows a logarithmic growth.

## Results

### General data of sTBI patients

In total, 20 patients with sTBI were enrolled in this study. There were 12 men and 8 women aged 32–56 years (median: 40 years).

### Quality control of the extracted RNA

The total RNA concentration was determined using NanoDrop2000C. The *A*_280_/*A*_260_ ratio was calculated to be 1.8–2.0, and the *A*_260_/*A*_230_ ratio was > 1.8. RNA integrity was estimated on an Agilent 2100 Bioanalyzer, and the RNA integrity number (RIN) was 10 for all samples. Therefore, the extracted RNA satisfied the requirements for miRNA microarray and real-time quantitative PCR.

### Identification of differential miRNA expression profiles in peripheral blood in sTBI patients

With the total RNA molecules extracted from all serum samples, a microarray was performed using a miscript miFinder Human Blood PCR Array kit, during which miRNA probes were labeled for neurological impairment. There were more than 84 capture probes for miRNA in the chip. The miRNA expression profiles were analyzed using miFinder miRNA PCR Array software, and the changes in the expression profiles of 84 miRNA molecules at 2, 12, 24, 48, and 72 h in the acute phase were dynamically observed.

**Fig. 1:**
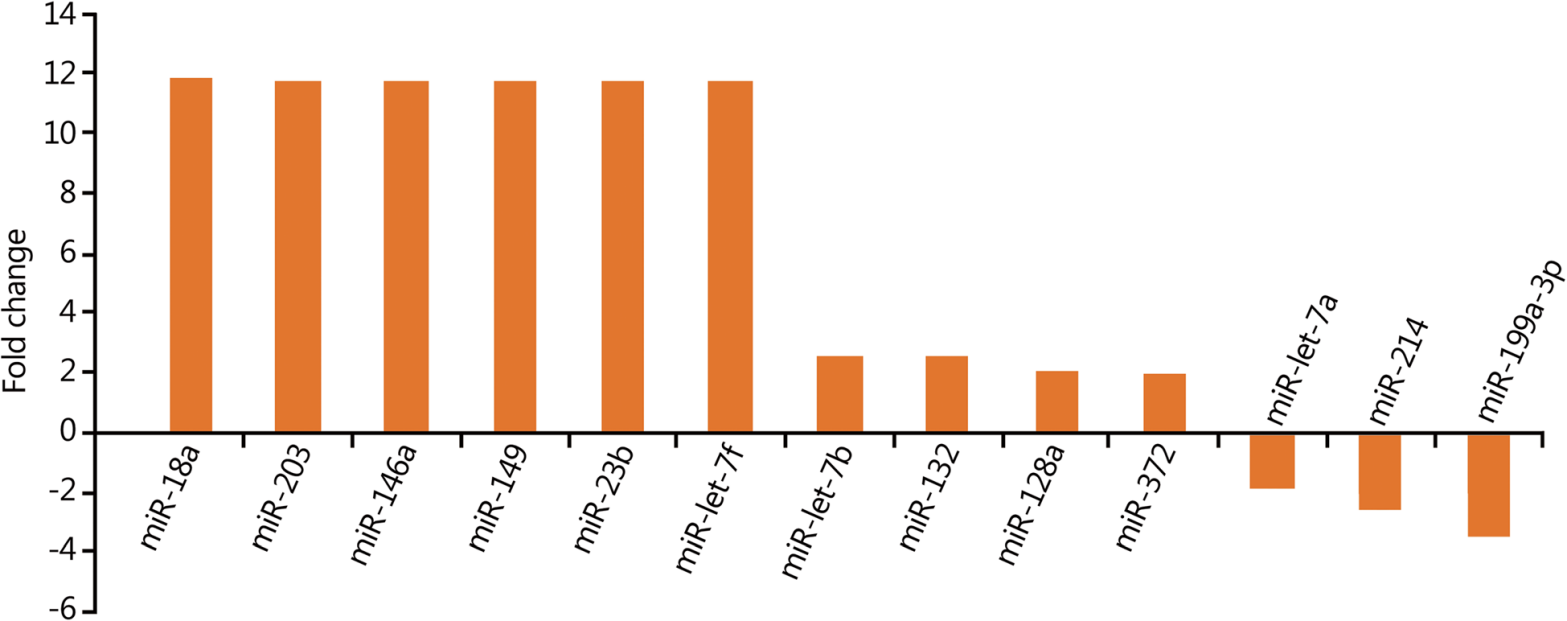
Changes in miRNA expression profiles in peripheral blood in the 12-h group compared with 2-h group

The expression levels of 10 miRNAs were upregulated more than 2-fold in the 12-h group compared with those in the 2-h group (Fig. 1). Among them, the expression levels of six miRNAs (miR-18a, miR-203, miR-146a, miR-149, miR-23b, and miR-let-7b) increased more than 10-fold (Fig. 1). The results of the target gene prediction are shown in Table 1.

**Table 1:** Main target genes of the differentially expressed miRNAs in the 12-h group compared with 2-h group

Change in miRNA expression	Name of miRNA	Predicted target genes
Upregulated	miR-18a	Neural precursor cell expressed; developmentally regulated protein 9; glycine receptor
	miR-203	4/all family, member 4; elongation factor RNA polymerase II, 2
	miR-146a	Zinc finger protein 826; tumor necrosis factor receptor-associated factor 6 (TRAF6)
	miR-149	AP2-associated protein kinase 1; PH domain and leucine-rich repeat protein phosphatase
	miR-23b	Zinc finger protein 138; fucosyltransferase (alpha-fucosyltransferase (1,3) 9
	miR-let-7f	High mobility group AT-hook 2; chromosome 14 open reading frame 28
	miR-let-7b	High mobility group AT-hook 2; chromosome 14 open reading frame 28
	miR-132	Transmembrane protein 106B; zinc finger protein 516
	miR-128a	Inflammatory pathways
	miR-372	Involved in DNA damage response
Downregulated	miR-let-7a	angiogenesis in rheumatoid arthritis via HIF-1α activation
	miR-214	NMDA receptor-regulated 2; κ-opioid receptor
	miR-199a-3p	Cadherin EGF LAG seven pass G-Type receptor 2; SH3 domain-containing GRB2-like endophilin B1

**Fig. 2:**
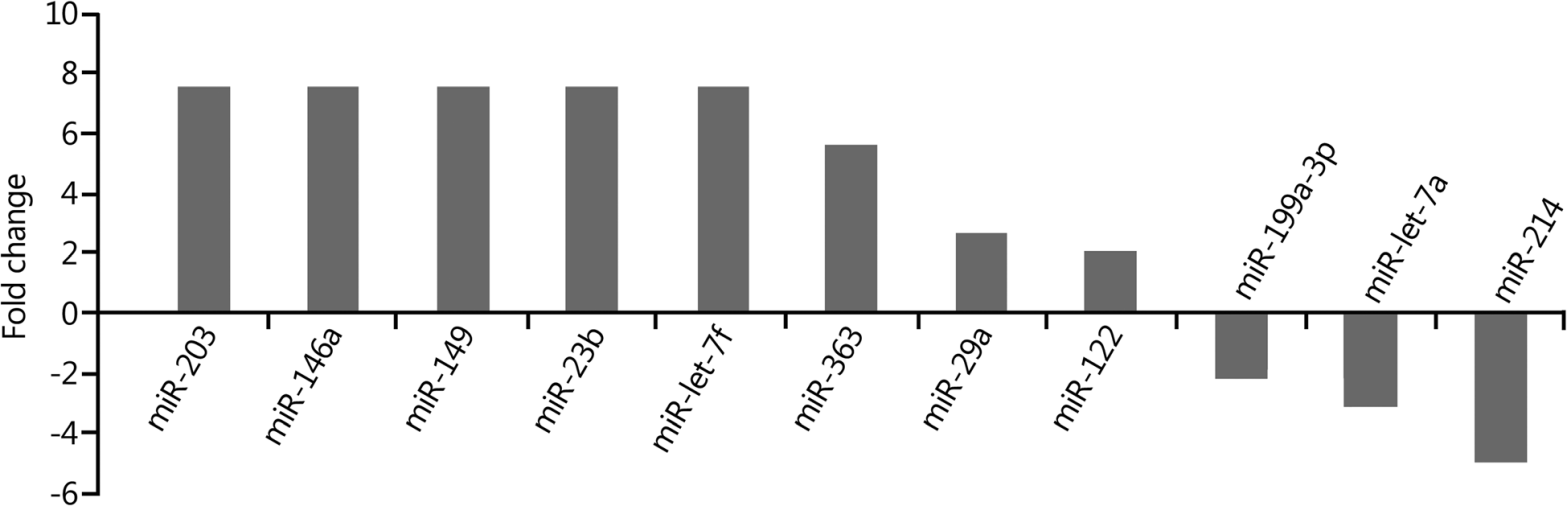
Changes in miRNA expression profiles in peripheral blood in the 24-h group compared with 2-h group

The expression levels of eight miRNAs in peripheral blood were upregulated more than 2-fold in the 24-h group compared with those in the 2-h group. Among them, the expression levels of five miRNAs (miR-203, miR-146a, miR-149, miR-23b and miR-let-7f) were increased more than 7-fold, and the expression levels of three miRNAs (miR-199a-3p, miR-let-7a, and miR-214) were decreased more than 2-fold (Fig. 2). The results of the target gene prediction are shown in Table 2.

**Table 2:** Main target genes of the differentially expressed miRNAs in the 24-h group compared with 2-h group

Change in miRNA expression	Name of miRNA	Predicted target genes
Upregulated	miR-203	4/all family, member 4; elongation factor RNA polymerase II, 2
	miR-146a	Zinc finger protein 826; tumor necrosis factor receptor-associated factor 6 (TRAF6)
	miR-149	AP2-associated protein kinase 1; PH domain and leucine-rich repeat protein phosphatase
	miR-23b	Zinc finger protein 138; fucosyltransferase (alpha-fucosyltransferase (1,3) 9
	miR-let-7f	High mobility group AT-hook 2; chromosome 14 open reading frame 28
	miR-363	Protects cardiomyocytes against hypoxia-induced apoptosis through regulation of Notch signaling
	miR-29a	ATPase family AAA domain-containing protein 2B; peptidase inhibitor 15
	miR-122	Chloride channel 4; regulatory factor X-associated protein
Downregulated	miR-199a-3p	Cadherin EGF LAG seven pass G-type receptor 2; SH3 domain-containing GRB2-like endophilin B1
	miR-let-7a	Angiogenesis in rheumatoid arthritis via HIF-1α activation
	miR-214	NMDA receptor-regulated 2; κ-opioid receptor

**Fig. 3:**
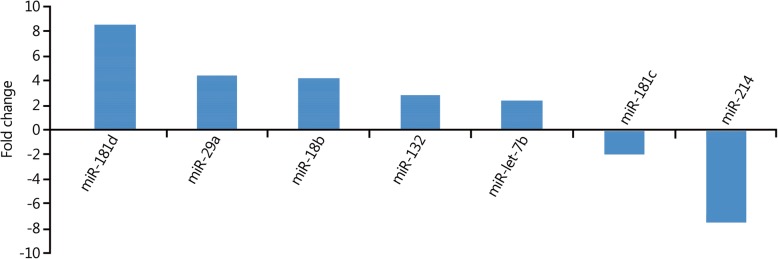
Changes in miRNA expression profiles in peripheral blood in the 48-h group compared with 2-h group

The expression levels of 5 miRNAs in peripheral blood were upregulated more than 2-fold in the 48-h group compared with those in the 2-h group. Among them, the expression levels of miR-181d increased more than 8-fold, and the expression levels of two miRNAs (miR-181C and miR-214) decreased more than 2-fold (Fig. 3). The results of the target gene prediction are shown in Table 3.

**Table 3:** Main target genes of the differentially expressed miRNAs in the 48-h group compared with 2-h group

Change in miRNA expression	Name of miRNA	Predicted target genes
Upregulated	miR-181d	Zinc finger protein 594; zinc finger protein 440
	miR-29a	ATPase family AAA domain-containing protein 2B; peptidase inhibitor 15
	miR-18b	Neural precursor cells expressed; developmentally regulated protein 9; glycine receptor
	miR-132	Transmembrane protein 106B; zinc finger protein 516
	miR-let-7b	High mobility group AT-hook 2; chromosome 14 open reading frame 28
Downregulated	miR-181c	Modulates the proliferation, migration, and invasion of neuroblastoma cells by targeting Smad7
	miR-214	NMDA receptor-regulated 2; κ-opioid receptor

**Fig. 4:**
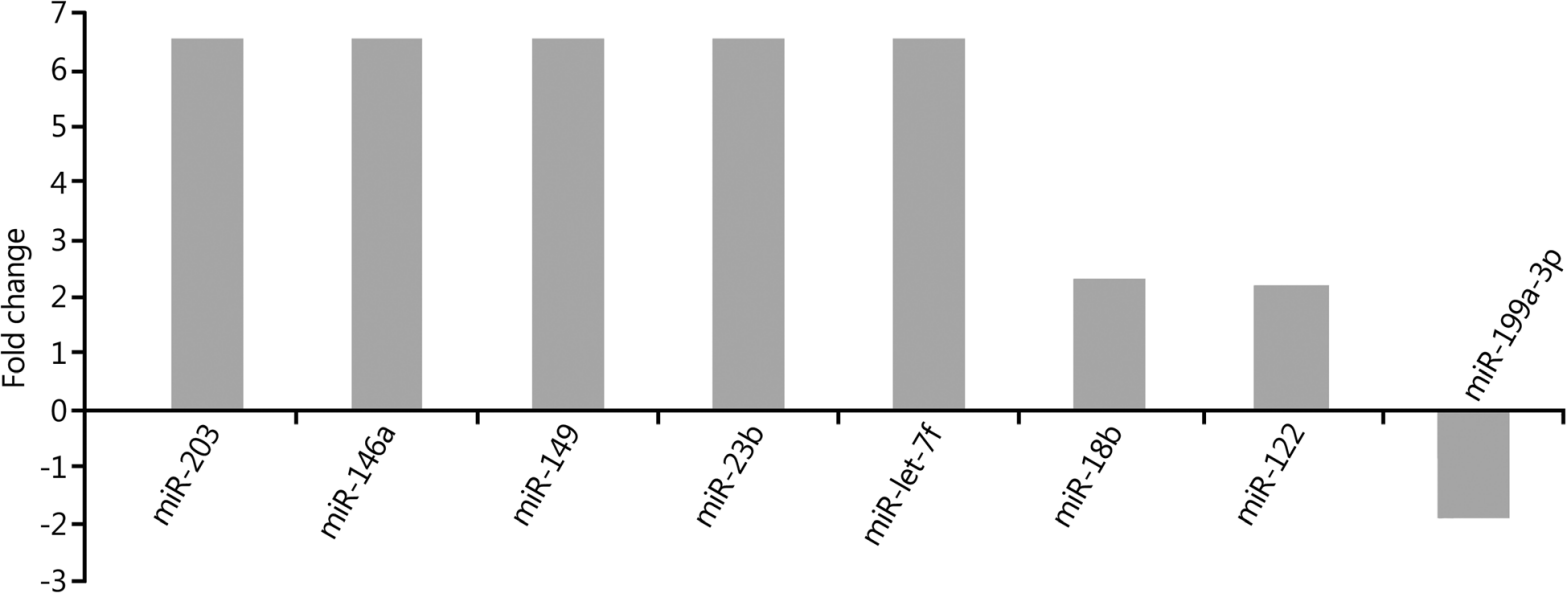
Changes in miRNA expression profiles in peripheral blood in the 72-h group compared with 2-h group

The expression levels of seven miRNAs in peripheral blood were upregulated more than 2-fold in the 72-h group compared with those in the 2-h group. Among them, the expression levels of five miRNAs (miR-203, miR-146a, miR-149, miR-23b and miR-let-7f) increased more than 5-fold, and the expression level of one miRNA (miR-199a-3p) decreased more than 2-fold (Fig. 4). The results of the target gene prediction are shown in Table 4.

**Table 4:** Main target genes of the differentially expressed miRNAs in the 72-h group compared with 2-h group

Change in miRNA expression	Name of miRNA	Predicted target genes
Upregulated	miR-203	4/all family, member 4; elongation factor RNA polymerase II, 2
	miR-146a	Zinc finger protein 826; tumor necrosis factor receptor-associated factor 6 (TRAF6)
	miR-149	AP2-associated protein kinase 1; PH domain and leucine-rich repeat protein phosphatase
	miR-23b	Zinc finger protein 138; fucosyltransferase (alpha-fucosyltransferase (1,3) 9
	miR-let-7f	High mobility group AT-hook 2; chromosome 14 open reading frame 28
	miR-18b	Neural precursor cells expressed; developmentally regulated protein 9; glycine receptor
	miR-122	Chloride channel 4; regulatory factor X-associated protein
Downregulated	miR-199a-3p	Cadherin EGF LAG seven pass G-type receptor 2; SH3 domain-containing GRB2-like endophilin B1

### Prediction of the target genes of differentially expressed miRNAs

Multiple miRNAs showed various changes in the peripheral blood in the 12-h, 24-h, 48-h, and 72-h groups compared with those in the 2-h group. The possible target genes of the differentially expressed miRNAs were predicted using TargetScan, miRanda, and PicTar software. It was found that, the neurological impairment following sTBI was mainly related to cell proliferation, apoptosis, differentiation, the inflammatory response, and collagen formation.

## Discussion

The sequelae of sTBI are complex and diverse [10, 11]. It has been recognized that the development of symptoms in the acute stage of TBI is associated with the following factors: inflammatory responses (e.g., serum response factors) [12], immune responses, vascular responses, and tumor necrosis factor [13]; DNA damage and repair; apoptosis; differential expression of cell growth factors; and pathways involving transcriptional regulatory proteins [14]. Research on miRNA expression profiles following TBI has suggested that miRNAs play a key role in this process [15]. A bioinformatics analysis showed that changes in miRNA expression affect the pathophysiological processes of brain injury, including secondary injury related to the inflammatory response and apoptosis [16]. Many studies on miRNA expression levels in patients with TBI have confirmed the changes in serum miRNA expression profiles in TBI patients [17].

In the present study, miRNA microarray and fluorescence-based quantitative real-time PCR were applied to detect and analyze the dynamic changes in miRNA expression levels in the peripheral blood of sTBI patients at high altitude. Our results showed that the expression levels of miR-18a, miR-203, miR-146a, miR-149, miR-23b, and miR-let-7b in peripheral blood increased more than 10-fold in the 12-h group compared with that of the 2-h group. These miRNA molecules might also be involved in the damage and repair of tumor necrosis factor (TNF) and DNA. In addition, the expression levels of miR-203, miR-146a, miR-149, miR-23b and miR-let-7f in peripheral blood increased more than 10-fold in the 24-h group. Similarly, both miR-203 and miR-let-7 were highly expressed in the acute phase, indicating that the inflammatory response was involved in the pathophysiological process of sTBI in high-altitude areas. Furthermore, the expression levels of miR-181d, miR-29a, and miR-18b increased more than 5-fold 48 h after the onset of sTBI at high altitude, and the expression levels of miR-203, miR-146a, and miR-149 increased over 5-fold after 72 h. Thus, the changes in the inflammatory response in the expression levels of miRNA molecules became mild compared with the acute phase response, and the differential genes gradually weakened, suggesting that the inflammatory response tended to be attenuated as the disease progressed. These dynamic changes in the miRNA expression profiles have provided important evidence to better understand the pathophysiological processes of sTBI at high altitude.

MiRNA is an important factor regulating inflammation. Yang et al. [18] suggested that microglia-mediated inflammation plays an important role in ischemic brain injury, while miR-203 negatively regulates ischemia-induced microglia activation by targeting MyD88. Through negative feedback, enforced expression of miR-203 silencing inhibits downstream NF-kappa beta signaling and microglia activation, thereby alleviating neuronal injury. The miR-let-7b mimetic reduces the expression of interleukin 6 (IL6) and TNF in monocytes and the expression of SERPINE1 in LPS-activated macrophages [19], which plays an anti-inflammatory role. Studies by Kumar et al. [20] showed that miR-let-7f overexpression reduced Mtb (*Mycobacterium tuberculosis*) survival and increased the production of cytokines such as TNF and interleukin 1 beta (IL-1β). Furthermore, miR-let-7f and its target A20 regulated the immune response to Mtb and controlled the bacterial load [20]. The role of miRNA is associated with TBI, and Sun et al. [21] found that overexpression of miR-23b conferred better neuroprotective effects after TBI by decreasing lesion volume, alleviating brain edema, inhibiting neuronal apoptosis and attenuating long-term neurological deficits. In addition, experimental animal studies have tested specific microRNAs such as miR-23b as biomarkers and therapeutic targets in cases of moderate and mild TBI [22]. These studies demonstrate, to varying degrees, the relationship between miRNAs found in this study and TBI or inflammation, enhancing the reliability of this study. Redell et al. [23] suggested that miR-16, miR-92a, and miR-765 were ideal biomarkers for severe brain injury, which is not entirely consistent with our findings. The possible explanation is that our subjects were sTBI patients from a high-altitude area.

## Conclusions

In summary, we observed dynamic differences in miRNA expression profiles in the peripheral blood of sTBI patients in the acute phase in a high-altitude area. Through the prediction of the main target genes of differentially expressed miRNAs, we could speculate about the pathophysiological evolution of sTBI and identify the miRNA expression levels related to the central nervous system in the acute phase of sTBI as well as miRNA expression levels at high altitude. Early interventions with drugs or other clinical therapies should be administered to achieve precise treatment, improve therapeutic efficacy, and reduce mortality and disability.

## Abbreviations

GCS: Glasgow Coma Scale
ICU: Intensive care unit
miRNAs: MicroRNAs
qRT-PCR: real-time quantitative PCR
RIN: RNA integrity number
sTBI: severe traumatic brain injury

## References

[1] Amantini A, Amadori A, Fossi S (2008). Evoked potentials in the ICU. Eur J Anaesthesiol Suppl.

[2] Amantini A, Carrai R, Lori S, Peris A, Amadori A, Pinto F (2012). Neurophysiological monitoring in adult and pediatric intensive care. Minerva Anestesiol.

[3] Bahloul M, Chelly H, Chaari A, Chabchoub I, Haddar S, Herguefi L (2011). Isolated traumatic head injury in children: analysis of 276 observations. J Emerg Trauma Shock.

[4] Arndt DH, Lerner JT, Matsumoto JH, Madikians A, Yudovin S, Valino H (2013). Subclinical early post-traumatic seizures detected by continuous eeg monitoring in a consecutive pediatric cohort. Epilepsia..

[5] Badjatia N (2009). Fever control in the neuro-ICU: why, who, and when?. Curr Opin Crit Care.

[6] Balakathiresan N, Bhomia M, Chandran R, Chavko M, Mccarron RM, Maheshwari RK (2012). Microrna let-7i is a promising serum biomarker for blast-induced traumatic brain injury. J Neurotrauma.

[7] Ballesteros MA, López-Hoyos M, Muñoz P, Marin MJ, Miñambres E (2007). Apoptosis of neuronal cells induced by serum of patients with acute brain injury: a new in vitro prognostic model. Intensive Care Med.

[8] Acharya SS (2015). Serum microRNAs are early indicators of survival after radiation-induced hematopoietic injury. Sci Transl Med.

[9] Bucy PC. Neurosurgical critical care. Surg Neurol. 1987;(1):80–1.

[10] Berardino M, Morrone O, Sciacca PF, Rosato R, Ciccone G, Massaro F (2004). Discharge criteria from intensive care unit in brain injured patients. Acta Neurochir.

[11] Corral L, Javierre CF, Ventura JL, Marcos P, Herrero JI, Mañez R (2012). Impact of non-neurological complications in severe traumatic brain injury outcome[J]. Crit Care.

[12] Bhalala OG. The Emerging Impact of microRNAs in Neurotrauma Pathophysiology and Therapy[M]// In: Kobeissy FH, editor. Brain Neurotrauma: Molecular, Neuropsychological, and Rehabilitation Aspects. Boca Raton (FL): CRC Press/Taylor & Francis; 2015. Chapter 26. 26269899

[13] Bryczkowski SB, Lopreiato MC, Yonclas PP, Sacca JJ, Mosenthal AC (2014). Risk factors for delirium in older trauma patients admitted to the surgical intensive care unit[J]. J Trauma Acute Care Surg.

[14] Dziurdzik P, Krawczyk L, Jalowiecki P, Kondera-Anasz Z, Menon L (2004). Serum interleukin-10 in ICU patients with severe acute central nervous system injuries. Inflamm Res.

[15] Ge XT, Lei P, Wang HC, Zhang AL, Han ZL, Chen X (2014). miR-21 improves the neurological outcome after traumatic brain injury in rats[J]. Sci Rep.

[16] Gim JA, Ha HS, Ahn K, Kim DS, Kim HS (2014). Genome-wide identification and classification of microRNAs derived from repetitive elements[J]. Genomics Inform.

[17] Greenberg JK, Stoev IT, Park TS, Smyth MD, Leonard JR, Leonard JC (2014). Management of children with mild traumatic brain injury and intracranial hemorrhage. J Trauma Acute Care Surg.

[18] Yang Z, Zhong L, Zhong S, Xian R, Yuan B (2015). miR-203 protects microglia mediated brain injury by regulating inflammatory responses via feedback to MyD88 in ischemia. Mol Immunol.

[19] Marques-Rocha JL, Garcia-Lacarte M, Samblas M, Bressan J, Martinez JA, Milagro FI (2018). Regulatory roles of miR-155 and let-7b on the expression of inflammation-related genes in THP-1 cells: effects of fatty acids. J Physiol Biochem.

[20] Kumar M, Sahu SK, Kumar R, Subuddhi A, Maji RK, Jana K (2015). MicroRNA let-7 modulates the immune response to mycobacterium tuberculosis infection via control of A20, an inhibitor of the NF-κB pathway. Cell Host Microbe.

[21] Sun L, Liu A, Zhang J, Ji W, Li Y, Yang X (2018). miR-23b improves cognitive impairments in traumatic brain injury by targeting ATG12-mediated neuronal autophagy. Behav Brain Res.

[22] Martinez B, Peplow PV (2017). MicroRNAs as diagnostic markers and therapeutic targets for traumatic brain injury. Neural Regen Res.

[23] Redell JB, Moore AN, Ward NH, Hergenroeder GW, Dash PK (2010). Human traumatic brain injury alters plasma microrna levels. J Neurotrauma.

